# Sea Level Budgets Should Account for Ocean Bottom Deformation

**DOI:** 10.1029/2019GL086492

**Published:** 2020-02-11

**Authors:** B. D. Vishwakarma, S. Royston, R. E. M. Riva, R. M. Westaway, J. L. Bamber

**Affiliations:** ^1^ School of Geographical Sciences University of Bristol Bristol UK; ^2^ Faculty of Civil Engineering and Geosciences Delft University of Technology Delft The Netherlands

**Keywords:** sea level budget, ocean bottom deformation, solid‐Earth response, GRACE, altimetry, steric

## Abstract

The conventional sea level budget (SLB) equates changes in sea surface height with the sum of ocean mass and steric change, where solid‐Earth movements are included as corrections but limited to the impact of glacial isostatic adjustment. However, changes in ocean mass load also deform the ocean bottom elastically. Until the early 2000s, ocean mass change was relatively small, translating into negligible elastic ocean bottom deformation (OBD), hence neglected in the SLB equation. However, recently ocean mass has increased rapidly; hence, OBD is no longer negligible and likely of similar magnitude to the deep steric sea level contribution. Here, we use a mass‐volume framework, which allows the ocean bottom to respond to mass load, to derive a SLB equation that includes OBD. We discuss the theoretical appearance of OBD in the SLB equation and its implications for the global SLB.

## Key Points

- The conventional sea level budget equation does not include elastic ocean bottom deformation, implicitly assuming it is negligible
- Recent increases in ocean mass yield global‐mean ocean bottom deformation of similar magnitude to the deep steric sea level contribution
- We use a mass‐volume approach to derive and update the sea level budget equation to include ocean bottom deformation as a process

## 1 Introduction

**Figure 1:**
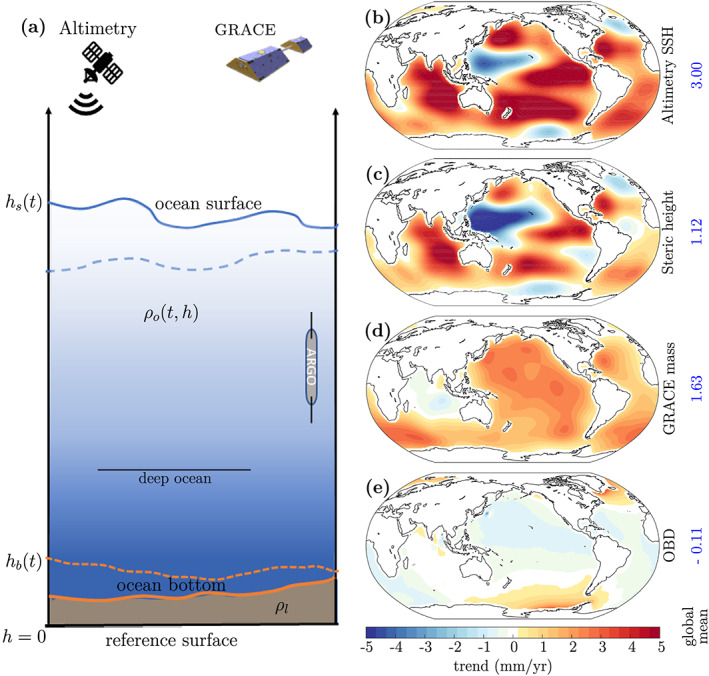
The sea level budget including ocean bottom deformation: (a) schematic diagram of a column of the Earth's solid and fluid envelope. The initial (dashed) and final (solid) state of the ocean bottom (orange) and sea surface (blue); (b) SSH trend from ESA SLCCI altimetry product; (c) steric sea level trend from ensemble mean of four steric data products; (d) ocean mass sea level trend from JPL GRACE Release 06 data; and (e) the OBD trend calculated from our approach. All the maps have been filtered with a 1,000‐km half‐width Gaussian filter and masked over land +300‐km buffer for visualization purposes.

Changes in sea surface height (SSH) can be explained by a combination of physical processes: the addition or removal of freshwater (mass change), change in ocean water volume (steric change), a change in ocean bottom topography (bathymetry), and ocean water redistribution driven by changes in the geoid and ocean circulation (cf. Figure 1a). Typically, solid‐Earth changes are assumed to be driven primarily by glacial isostatic adjustment (GIA; herein, we only consider the viscous response of the solid Earth to deglaciation after the Last Glacial Maximum). Several GIA forward models are available and are used as a correction to SSH observations. Thus, conventional sea level budget (SLB) studies equate total SSH change to a sum of mass and steric sea level change (Bindoff et al., 2007; Leuliette & Willis, 2011). As a result, SLB studies are essential to understand the temporal evolution of different contributors to contemporary sea level rise.

The global‐mean SLB is reported to close within the uncertainties of the observation systems, when taking an ensemble mean of observational products available from different sources (WCRP, 2018). However, there can be large differences in the trends and spatial patterns from different sets of observational data products (Chambers et al., 2017; Dieng et al., 2015; WCRP, 2018), and the SLB at the ocean‐basin scale does not close (Dieng et al., 2015; Llovel et al., 2010; Purkey et al., 2014). A gap in the SLB can be attributed to inaccuracies or poor sampling in the observations and/or neglecting physical processes in the budget equation. Therefore, identifying individual processes and understanding how their magnitude is changing in time is important to improve sea level forecasting abilities. For example, there is increasing concern over the uncertainty in magnitude of the deep steric contribution to sea level change (that below 2,000 m not measured by the ARGO float network; Dieng et al., 2015; Purkey et al., 2014; WCRP, 2018), which motivated the community to invest in deep ARGO floats. However, elastic deformation of the ocean bottom due to changes in present‐day mass loading has not received as much attention.

It is known that the solid‐Earth responds instantaneously and elastically to changes in the surface mass load (Farrell, 1972). There is clear evidence that sea level rise, which was dominated by thermosteric change during the 20 th Century, is now dominated by ocean‐land mass exchange (Bamber et al., 2018; Chen et al., 2017; WCRP, 2018). The small ocean mass increase during the last Century meant that elastic ocean bottom deformation (OBD) was significantly smaller than uncertainties and it could safely be ignored in the SLB equation. However, the contemporary acceleration in ocean mass makes OBD nonnegligible. Recently, it has been shown that the theoretical elastic OBD due to changes in mass load since 1993 contributes approximately 0.13 mm/year (or 3–4%) to global‐mean sea level change (Frederikse et al., 2017). This contribution is comparable in magnitude to the deep steric contribution, which has been identified as one of the top priorities in sea level research (Roemmich et al., 2019; WCRP, 2018). Due to ongoing global warming, the rate of ocean mass change is expected to continue to increase, which will in‐turn increase the elastic OBD in the near future.

In the SLB community, several different methods are employed to estimate mass sea level change from observations. Ocean mass changes may be directly observed over the oceans (as used by Chambers et al., 2004; Fenoglio‐Marc et al., 2006; Llovel et al., 2010) or may be indirectly determined invoking the conservation of mass for the whole Earth system, where ocean mass is the sum of contributions from land mass changes (e.g., Dieng et al., 2017; WCRP, 2018). To obtain a realistic spatial distribution of the indirect ocean mass, gravitation, rotation, and deformation effects are incorporated via the sea level equation (Farrell & Clark, 1976), resulting in “sea level fingerprints” (Adhikari et al., 2019; Frederikse et al., 2017; Riva et al., 2010). We note these latter sea level fingerprints intrinsically account for OBD as part of the gravitation, rotation, and deformation component, but not ocean mass redistribution (manometric changes) due to other processes such as wind stress and ocean circulation changes.

A small number of studies acknowledge the OBD component of sea level and recommend including it as a correction to the absolute SSH anomaly observations from satellite altimetry or intrinsically in sea level fingerprints derived from land mass change observations (Frederikse et al., 2017; Kuo et al., 2008; Ray et al., 2013). Yet OBD is still not included in SLB studies that use direct GRACE ocean mass change estimates or the simple sum of mass change estimates over land (WCRP, 2018). Since many studies use the SLB equation as a constraint to assess the quality of a time series or to estimate one component from the residual of the SSH minus the other budget components, updating the SLB equation, so that it accurately represents processes contributing to sea level change, is vital.

In this paper we first discuss the conventional SLB and then derive an updated SLB equation from a mass‐volume approach. The updated SLB equation has a dedicated term for OBD along with steric and mass terms. We show that the new SLB equation is equivalent to the conventional SLB equation under the assumption that the elastic deformation of the ocean floor due to ocean mass change is negligible. Our updated SLB equation is then applied using satellite altimetry SSH anomaly, GRACE ocean mass, in situ measured steric data, and an estimate of OBD in the updated SLB equation. We obtain our estimate of OBD from GRACE‐observed mass redistribution and elastic load Love numbers for a PREM solid‐Earth model (Dziewonski & Anderson, 1981). We discuss implications of this updated budget equation for SLB studies using various data products.

## 2 The SLB Equation

### 2.1 The Conventional SLB

In the contemporary SLB equation, a change in SSH is compared with the sum of mass and steric sea level components (Bindoff et al., 2007; Chambers et al., 2004, 2017; Church et al., 2013; Dieng et al., 2015, 2017; Fenoglio‐Marc et al., 2006; Leuliette & Miller, 2009; Llovel et al., 2010; WCRP, 2018):

(1) $$\dot{\eta }={\dot{\eta }}_{m}+{\dot{\eta }}_{s}+\epsilon ,$$

where $\dot{\eta }$ denotes rate of change of SSH, ${\dot{\eta }}_{m}$ is the rate of change of ocean‐mass sea level, ${\dot{\eta }}_{s}$ is the rate of change of steric sea level, and ϵ is the difference often described as the misclosure.

Comparison of observed changes in these components has led to numerous studies, since the “sea level enigma” of Munk (2002), comparing independently observed mass, steric, and total sea level changes (see WCRP, 2018, and references therein). The total sea level change is typically represented by SSH derived from satellite altimetry, which must also be corrected for vertical land movement typically assumed to be predominantly due to GIA. Several GIA forward models are available, but in practice, SLB studies conventionally adopt a standard global‐mean correction of −0.3 mm/year (Tamisiea, 2011; WCRP, 2018). The ocean mass change is determined either from GRACE fields over the ocean or from estimates of changes in water mass stored over land in ice sheets, glaciers, and liquid water bodies (Chambers et al., 2017; Frederikse et al., 2017; Rietbroek et al., 2016; WCRP, 2018). Since GRACE observes total mass change near the Earth's surface, ocean mass change is obtained by removing the GIA‐induced mass change from GRACE products. Steric sea level change is typically determined from products that incorporate ARGO floats, drifting or moored profiles and ship‐bourne profiles (Church et al., 2011; Gaillard et al., 2016; WCRP, 2018). Since the ARGO floats, until very recently, only measured the upper 2,000 m of the oceans, typically an estimate of deep‐ocean steric sea level change from other sources is included in a full‐depth estimate of steric change.

The elastic deformation component is not accounted for by satellite altimetry measurements. Frederikse et al. (2017) show that there is a mismatch between observations of SSH from satellite altimetry and the sum of mass and steric sea level change owing to this contemporary elastic change to the ocean bottom (cf. Figure 1a).

### 2.2 A Mass‐Volume Approach

We derive a SLB equation using the law of conservation of mass and the mass‐volume relation. Imagine a reference surface h=0 below the ocean's deepest point. We want to calculate the total mass between this reference and a point above the ocean surface (Figure 1a). The density of ocean water varies spatially, with height and in time due to changes in temperature and salinity; thus, we represent it as a function of latitude λ, longitude θ, height h, and time t: $\rho_{o}(\lambda ,\theta ,h,t)$. $\rho_{atm}(\lambda ,\theta ,h,t)$ is the density of the atmosphere, and the global‐mean density of the solid Earth is represented by $\rho_{l}$. The ocean surface and the ocean bottom varies with time; thus, we can represent them as $h_{s}(t)$ and $h_{b}(t)$, respectively. The total mass M of a column at time t can be written as

(2) $$\begin{array}{ccc} M(t) & =\int_{\theta }\int_{\lambda }\int_{h}\rho \;dv & \\ & =\int_{\theta }\int_{\lambda }\int_{0}^{h_{b}(t)}\rho_{l}\;dv+\int_{\theta }\int_{\lambda }\int_{h_{b}(t)}^{h_{s}(t)}\rho_{o}(\lambda ,\theta ,t,h)\;dv+\int_{\theta }\int_{\lambda }\int_{h_{s}(t)}^{h_{atm}}\rho_{atm}(\lambda ,\theta ,t,h)\;dv. \end{array}$$

Over decadal time scales, change in the spatial extent of the ocean is very small compared to the total area, so for the global mean we ignore time‐varying spatial extent of oceans in latitude and longitude. Thus, $\int_{\theta }\int_{\lambda }\int_{h}dv$ reduces to $a\int_{h}dh$, $\rho_{o}(\lambda ,\theta ,t,h)$, and $\rho_{atm}(\lambda ,\theta ,t,h)$ reduces to the spatial mean ocean density $\rho_{o}(t,h)$ and the spatial mean atmospheric density $\rho_{atm}(t,h)$, respectively, that varies only in height and time (Figure 1). Differentiating equation (2) with respect to time, we get the rate of mass change:

(3) $$\frac{dM}{dt}=a\;\frac{d}{dt}\int_{0}^{h_{b}(t)}\rho_{l}\;dh+a\;\frac{d}{dt}\int_{h_{b}(t)}^{h_{s}(t)}\rho_{o}(t,h)\;dh+a\;\frac{d}{dt}\int_{h_{s}(t)}^{h_{atm}(t)}\rho_{atm}(t,h)\;dh.$$

In analogy with the conventional SLB equation, we wish to separate the mass and steric components of sea level change. Using the Leibniz integral rule, we can rewrite the ocean density integral in equation (3)as

(4) $$\frac{d}{dt}\;\int_{h_{b}(t)}^{h_{s}(t)}\rho_{o}(t,h)\;dh=\rho_{o}(t,h_{s}(t))\frac{dh_{s}(t)}{dt}-\rho_{o}(t,h_{b}(t))\frac{dh_{b}(t)}{dt}+\int_{h_{b}(t)}^{h_{s}(t)}\frac{\partial \rho_{o}(t,h)}{\partial t}dh.$$

For simplicity hereafter, we will denote the ocean surface and ocean bottom densities $\rho_{o}(t,h_{s}(t))=\rho_{o}^{s}(t)$ and $\rho_{o}(t,h_{b}(t))=\rho_{o}^{b}(t)$.

Equation (4) therefore separates the change in volume of the ocean into mass changes (the first and second terms on the right‐hand side) and volume changes due to changes in density in time (the last term).

#### 2.2.1 A Revised SLB From Observations

The first term on the right‐hand side of (3)represents the solid‐Earth mass redistribution. A change in the ocean bottom occurs due to both the viscous and the elastic response of the solid Earth to the mass load change. The elastic deformation occurs instantaneously under the influence of contemporary mass load on the surface of the Earth, and it affects the lithosphere more than the rest of the mantle (Farrell, 1972; Han & Wahr, 1995). On the other hand, the viscous deformation induces mass redistribution due to a slow influx of mantle material, as a response to all the surface mass load changes since the Last Glacial Maximum (Geruo et al., 2013; Han & Wahr, 1995). There is a radial component of solid‐Earth deformation as well as a small horizontal component, which are modeled by forward GIA models (van Dam et al., 2007; Wahr et al., 2013). Therefore, we rely on a forward GIA model for an estimate of the first right‐hand term in equation (3).

Nominally, the rate of change in mass, the left‐hand side of equation (3), is determined from GRACE, a gravimetry mission that observes mass movement within and between the solid‐Earth, ocean, and atmosphere, at a native resolution of approximately 300 km (Tapley et al., 2004; Vishwakarma et al., 2018). GRACE measures the total mass change, and to obtain ocean mass change estimates, we remove the GIA and high‐frequency atmospheric mass changes:

(5) $$\frac{dM}{dt}-a\;\frac{d}{dt}\int_{0}^{h_{b}(t)}\rho_{l}\;dh-a\;\frac{d}{dt}\int_{h_{s}(t)}^{h_{atm}}\rho_{atm}(t,h)\;dh\approx \frac{dM_{o}}{dt},$$

where $\frac{dM_{o}}{dt}$ is the rate of ocean mass change deduced from GRACE. Changes in gravitational field observed by GRACE are converted to mass change in mm of equivalent water height (EWH) assuming freshwater density, so we can write the rate of ocean mass change as the rate of change given by GRACE in EWH multiplied by freshwater density $\rho_{w}$ and the area a:

(6) $$\frac{dM_{o}}{dt}=a\rho_{w}\frac{d{\hat{\eta }}_{m,\text{EWH}}}{dt}.$$

Here, we denote the observation of ocean mass from GRACE (as EWH and corrected for GIA and atmospheric variability) as ${\hat{\eta }}_{m,\text{EWH}}$.

Changes in SSH are derived from satellite altimetry, here denoted $\hat{\eta }$, which when multiplied by the ocean surface density $\rho_{o}^{s}(t)$ of the water column a gives us the first term on the right‐hand side of (4):

(7) $$\rho_{o}^{s}(t)\frac{dh_{s}(t)}{dt}\approx \rho_{o}^{s}(t)\frac{d\hat{\eta }}{dt}.$$

Similarly, the derivative, $\frac{dh_{b}(t)}{dt}$, on the right‐hand side of (4) denotes the (unobserved) rate of change in the ocean bottom $h_{b}$, which represents elastic OBD.

The final term on the right‐hand side of equation (4) represents the change in steric sea level in time (see supporting information S2). We denote the observed steric sea level by ${\hat{\eta }}_{s}$.

(8) $$\int_{h_{b}(t)}^{h_{s}(t)}\frac{\partial \rho_{o}(t,h)}{\partial t}dh\approx -{\bar{\rho }}_{o}(t)\frac{d{\hat{\eta }}_{s}}{dt},$$

where ${\bar{\rho }}_{o}(t)$ is the area‐mean depth‐averaged ocean water density in that column. Substituting equations 7 and (8) in (4) gives the change in ocean mass in terms of purely volume and mass‐exchange terms. Substituting assumptions (5) and (6) with the ocean mass into the full mass‐volume derived equation (3)and rearranging for $\hat{\eta }$ gives

(9) $$\frac{d\hat{\eta }}{dt}\approx \frac{1}{\rho_{o}^{s}(t)}\left\{\rho_{w}\frac{d{\hat{\eta }}_{m,\text{EWH}}}{dt}+{\bar{\rho }}_{o}(t)\frac{d{\hat{\eta }}_{s}}{dt}+\rho_{o}^{b}(t)\frac{dh_{b}}{dt}\right\}.$$

This is the updated SLB equation. Note that the steric part in equation (9) is for full ocean and it also contains deep‐steric contributions. The last term represents OBD.

The revised SLB equation reduces to the conventional SLB if we assume that the elastic response of the ocean bottom is negligible, that is, $h_{b}(t)$ is time invariant, in which case the third term in the right‐hand side of (9) will become zero. Conventionally, $\rho_{o}^{s}(t)$ and $\rho_{o}^{b}(t)$ are assumed to be equal to average ocean density ${\bar{\rho }}_{o}$. Applying these constraints, we get

(10) $$\frac{d\hat{\eta }}{dt}\approx \left\{\frac{\rho_{w}}{{\bar{\rho }}_{o}}\frac{d{\hat{\eta }}_{m,\text{EWH}}}{dt}+\frac{d{\hat{\eta }}_{s}}{dt}\right\}.$$

Note that many budget studies compare altimetry height changes to a sum of GRACE freshwater height changes and steric height changes, while a few use ocean water density to estimate GRACE EWH over the oceans (Chambers et al., 2004; Dieng et al., 2015; Fenoglio‐Marc et al., 2006; Rietbroek et al., 2016). This choice leads to a 3% difference in GRACE estimates. As shown in equation (10), we should either estimate EWH from GRACE using ocean water density or we should scale the GRACE freshwater EWH before using them in the budget equation. In the next section, we compare our revised SLB equation with the conventional approach.

## 3 Applying the Updated Budget Equation

We compare the revised SLB equation with the conventional equation between January 2005 and December 2015 inclusive, as this period covers the best quality data with maximum spatial coverage from ARGO floats and GRACE gravimetry measurements.

We use SSH from satellite altimetry, steric heights from temperature and salinity data, and GRACE‐observed ocean mass. In order to be consistent, we process the available data to obtain monthly time series at 1° grid cells for the period between January 2005 and December 2015. An area‐weighted mean of grid scale time series over oceans is computed for each quantity, where we mask out continents and regions within 300 km from coastline to minimize land‐ocean leakage signal (Chambers et al., 2017; Church et al., 2013). The global‐mean time series of individual components is shown in Figure S1. Each time series is then decomposed into an intercept, a linear trend, a semiannual signal, and an annual signal using least squares regression. One standard deviation of the residual between the least squares fit and the data is used to denote the uncertainty of the fit.

### 3.1 Altimetry‐Derived SSH

We use SSH time series compiled from multimission altimetry at 0.25° grid resolution, which is provided by the European Space Agency's sea level climate change initiative (SLCCI) project Version 2.0 (Ablain et al., 2015; ESA, 2018; Legeais et al., 2018; Quartly et al., 2017). The global product provides an anomaly from the mean sea surface in the geocentric frame of reference and includes corrections for atmosphere perturbations, instrument error, ocean tide, solid‐Earth tide, and pole tide (including linear pole; Desai et al., 2015). The SSH product is also corrected for the mean impact of GIA over oceans by adding a constant value of −0.3 mm/year (Argus et al., 2014; Peltier et al., 2015). We remove the same GIA altimetry correction from ICE6G_D‐VM5a spatially at grid scale to ensure consistent GIA correction between altimetry and GRACE, from spherical harmonics (Peltier, 2018). A global map of the smoothed altimetry SSH trend is shown in Figure 1b.

### 3.2 Steric Height Change

We use temperature and salinity profiles from four research groups to compute steric sea level change by considering the Thermodynamic Equation Of Sea water as the equation of state (http://www.teos-10.org) (IAPWS, 2008; Millero et al., 2008). The data sets we use here are from Scripps Institution of Oceanography (Roemmich & Gilson, 2009; Scripps Oceanographic Institute, 2018); Japan Agency for Marine‐Earth Science and Technology (Hosoda et al., 2008; JAMSTEC, 2018); UK Met Office's EN4.2.1 model (Good et al., 2013; Gouretski & Reseghetti, 2010; UKMO, 2018); and ISAS13 from IFREMER (Gaillard et al., 2016; IFREMER, 2018).

Scripps Institution of Oceanography data use temperature and salinity measurements from Argo floats only, while the other three data sets supplement the ARGO float data with in situ measurements. The temperature and salinity at standard depths are converted to a steric sea level change from monthly mean climatology and interpolated up the water column from 2,000 m to the surface. Monthly anomaly time series are zero‐meaned. The ensemble mean of these four SSL time series is then used in the calculation of the global mean steric sea level time series. The steric data are a good representative of oceans to a depth of 2,000 m only. Therefore, we add the deep steric component to the global mean separately, taking an estimate of the global‐mean deep steric correction as 0.1 mm/year (Church et al., 2011; WCRP, 2018). A global map of smoothed steric sea level trend is shown in Figure 1c.

### 3.3 GRACE‐Observed Ocean Mass Change

We use the JPL Release 06 GRACE mascon data because they have been shown to match the in situ ocean bottom pressure (Watkins et al., 2015; Wiese et al., 2018). One of the differences between Releases 05 and 06 is the improved Atmosphere Ocean Dealiasing model (AOD1B), which improves product quality over oceans (Dobslaw et al., 2017; Göttl et al., 2018; Wiese et al., 2018). Since the native resolution of GRACE products is around 3° (Tapley et al., 2004; Vishwakarma et al., 2018; Watkins et al., 2015), the coastal ocean is affected by land‐ocean signal leakage (Chambers et al., 2004). Therefore, it is prudent to avoid coastal regions prior to calculations (Chambers et al., 2004, 2017); we use a 300‐km buffer around land. The JPL mascon products have been treated for GIA by removing modeled estimates also from ICE‐6G forward GIA model (Peltier et al., 2015). We restore the oceanic component of the AOD1B dealaising products to obtain full ocean mass (Dobslaw et al., 2017). A global map of smoothed GRACE ocean mass trend is shown in Figure 1d.

### 3.4 OBD From Loading Theory

We use mass change rates from GRACE and the elastic loading Love numbers to obtain vertical deformation of the Earth's surface. First, we convert the trend field from GRACE to gravitational spherical harmonic coefficients, which are used in the synthesis equation (van Dam et al., 2007):

(11) $$\dot{r}(\theta ,\lambda )=R\underset{l,m}{\sum }{\tilde{P}}_{lm}[{Ċ}_{lm}\cos(m\lambda )+{\dot{S}}_{lm}\sin(m\lambda )]\frac{h_{l}^{\prime }}{1+k_{l}^{\prime }},$$

where $\dot{r}$ is the rate of displacement of the Earth's surface in the radial direction, $h_{l}^{\prime }$ and $k_{l}^{\prime }$ are the load Love numbers of degree l, $P_{lm}$ is normalized Legendre functions of degree l and order m, and ${Ċ}_{lm}$ and ${\dot{S}}_{lm}$ represent the rate of change in Stokes coefficients of mass. The load Love numbers used to transform mass coefficients into estimates of crustal deformation are obtained from Han and Wahr (1995). The rate of vertical crustal deformation obtained is a global field, which is then limited to oceans, by masking out land and a 300‐km buffer, to get the OBD trend field. The global pattern of OBD is shown in Figure 1e. As expected, we observe that the ocean bottom has subsided where the ocean mass is increasing.

### 3.5 Closing the SLB Equation

For the period January 2005 to December 2015 inclusive, and with our masking of land plus a 300‐km buffer, the ESA SLCCI altimetry product gives a global‐mean SSH trend rate of 3.00 ± 0.12 mm/year. GRACE detects an increase in the ocean mass sea level at a rate of 1.63 ± 0.10 mm/year. Our ensemble mean steric sea level gives a rate of 1.12 ± 0.06 mm/year (Table 1). We estimate OBD from loading theory to be −0.11 ± 0.02 mm/year. Our ensemble mean steric sea level change does not contain a deep steric contribution, so we use an estimate of the global‐mean deep steric correction: 0.1 mm/year (Church et al., 2011; WCRP, 2018). The uncertainty in GIA models and its inconsistent application to altimetry and gravity products (Tamisiea, 2011) are a source of SLB misclosure. We have ensured a consistent treatment of GIA for GRACE and altimetry, which reduces its impact on the SLB. Furthermore, we mask out continents and nearby coast, which removes regions with large GIA signal and land‐ocean leakage in GRACE. Solving the conventional SLB equation (1) leaves us with a gap of 0.15 mm/year (cf. Table 1). However, when using the updated SLB equation, this gap increases to 0.26 mm/year. Compared with the recent review from WCRP (2018) of the conventional SLB, it is noted that the rate of change estimates calculated here differs from the published WCRP (2018) trend rates for a number of reasons, such as time period of the study and processing choices. We recalculate the trend values for WCRP to match the time period of this study (Table 1). The remaining differences are of the order of 0.1 mm/year in the global mean from sources including the choice of data products and their corrections (in particular the choice of GIA correction and the fact WCRP take ensemble means of data products), the latitudinal extent and the land/buffer mask chosen, and the method of trend calculation. While these choices lead to differing absolute trend values for SSH, mass, and steric sea level change, the SLB gap is of approximately the same magnitude (cf. Table 1).

**Table 1:** Linear Trends in Sea Level Components Between 2005 and 2015, for This Study and for WCRP Study

Study	SSH	Steric	Mass	OBD	Gap (SLB)
Conventional SLB (this study)	3.00±0.12	1.22±0.06	1.63±0.10	—	0.15
Updated SLB (this study)	3.00±0.12	1.22±0.06	1.63±0.10	−0.11	0.26
WCRP	3.5±0.2	1.4±0.4	2.0±0.19	—	0.1
WCRP (with OBD)	3.5±0.2	1.4±0.4	2.0±0.19	−0.1	0.2

*Note.* We have calculated trends from WCRP time series to match the time period.

OBD is estimated using global mass change fields from the JPL GRACE mascon product with AOD1B dealiasing models for atmospheric and ocean mass change restored. Since GRACE mass change estimates from different centers vary due to the specific processing strategy and choice of $C_{20}$ and degree 1 coefficients, using a different GRACE product is expected to yield a different OBD. Furthermore, the elastic solid‐Earth vertical uplift due to present‐day mass loss is largest near the coast. If the global‐mean SLB were calculated without a 300‐km buffer, the global‐mean OBD would be affected. We assess the sensitivity of OBD with respect to the change in GRACE product, and application of buffer and find global‐mean OBD is negative for all such estimates with the same order of magnitude (Figure S2 and Table S1). Thus, our OBD estimate is robust. Frederikse et al. (2017) used mass change estimates over land to estimate total mass change over oceans. This mass was distributed over the oceans using the sea level equation in a gravitationally consistent manner. The mass change pattern was then used to calculate an OBD rate of −0.13 ± 0.01 mm/year between 1993 and 2014. For the period 2005 to 2014, the OBD rate from the data published by Frederikse et al. (2017) is −0.13 mm/year for the entire global ocean and −0.21 mm/year when applying a 300‐km land buffer. The global mean without the buffer is a smaller quantity because the uplift near the coast of Greenland and Antarctica, due to present‐day mass loss, negates the subsidence elsewhere.

## 4 Summary and Conclusion

In this study we revised the SLB equation from a mass‐volume approach. We apply appropriate approximations and assumptions to develop a SLB equation that is consistent with contemporary sea level processes and existing observational data sets. We build on recent studies to show that the global‐mean change in OBD is significant and similar in magnitude to the estimate of deep steric change.

Since direct observation of ocean bottom elevation is not feasible, we rely on elastic Love load numbers and GRACE products for obtaining an estimate of OBD. Using our updated SLB equation, the gap in the SLB is found to increase. This calls for a careful reassessment of SLB studies that have previously ignored OBD.

Our ability to predict future sea level is constrained by our understanding of contemporary sea level variability and its driving processes. Therefore, many attempts have been made at closing the SLB that equates changes in SSH to a sum of mass and steric sea level change. This equation was repeatedly used in the late 1990s when it was assumed that the elastic deformation of ocean bottom due to ocean mass change was negligible. Sea level rise in the 20th Century was considered to be dominated by steric change, but in recent decades, the mass component has become more significant meaning that the elastic OBD should no longer be considered negligible and must be accounted for. We recommend that our updated SLB should be used by any Earth Scientist either trying to close the SLB or using the SLB equation to test the robustness of new data products or methods. Our revised equation will facilitate these objectives and will help ensure that OBD can be correctly acknowledged as an increasingly important sea level process.

## Supporting information



Supporting Information S1

Supporting Information S2

Figure S1

Figure S2
