# Supplementary material for: Sea Level Budgets Should Account for Ocean Bottom Deformation
Source: Geophys Res Lett. 2020 Feb 11;47(3):e2019GL086492. doi: 10.1029/2019GL086492 (PMC7687171; doi:10.1029/2019GL086492)
Supplement: Supplementary file 1 — Supporting Information S1 [file GRL-47-e2019GL086492-s001.pdf]

Supporting Information for "Updating the Sea Level Budget Equation"

B. D. Vishwakarma [1], S. Royston [1], R. E. M. Riva[2], R. M. Westaway[1], J. L. Bamber [1]

[1]School of Geographical Sciences, University of Bristol, UK

[2]Faculty of Civil Engineering and Geosciences, Delft University of Technology, The Netherlands

Contents of this file

1. Text S1 to S2
2. Figures S1
3. Tables S1

Text S1: Ocean Bottom Deformation from Different GRACE Products

We use GRACE mascon products from JPL, CSR, and GSFC **????**, and spherical harmonic products from CSR, GFZ and ITSG **???**. The recommended post-processing steps for obtaining mass change from spherical harmonic coefficients were followed: we first replaced the C_{20} and degree 1 coefficients **??**, and then subtracted a mean static gravity field (computed from full GRACE coefficients between January 2004 and December 2009) to obtain residual coefficients. These were filtered with a destriping filter and a Gaussian filter of half width radius of 300 km and converted to mass anomaly in terms of equivalent water height **?**. In or-

der to restore the monthly mean ocean mass signal removed during de-aliasing, we restored GAB product to the monthly GRACE fields **?**.

All of the GRACE products were then used to obtain trends at 1° grid scale, which were then used in equation (11) to obtain a global field of solid Earth elastic deformation due to changes in mass load, which is then be masked to get elastic deformation over oceans only (we may exclude oceans within 300 km of the coastline by adding a buffer) and its area weighted mean gives us mean OBD. Please note that the GRACE mass change from each product is different and so is the corresponding OBD (Figure S1 and Table S1).

Text S2: Steric Sea Level

By definition, $p = \rho g h$ where p is pressure at a given location and time. In the case of hydrostatic equilibrium, at a constant pressure level:

$$p_{t_0} = \rho_{t_0} g h_{t_0};$$

$$p_{t_1} = \rho_{t_1} g h_{t_1};$$

$$\therefore \rho_{t_0} g h_{t_0} = \rho_{t_1} g h_{t_1}$$

A change in density between two time steps at a constant pressure level and with no change in mass will invoke a change in height in a water column due to steric changes, $\Delta\eta_s$:

$$\begin{aligned}\Delta\eta_s &= h_{t_1} - h_{t_0} \\ &= \frac{\rho_{t_0} - \rho_{t_1}}{\rho_{t_1}} h_{t_0} \\ &= -\frac{\Delta\rho_o}{\rho_{t_1}} (h_s(t_0) - h_b(t_0))\end{aligned}$$

The observed steric sea level products give a best estimate at the discretised spatial rate of change in steric sea level $\Delta\eta_s$ from temperature and salinity measurements throughout the

water column, $h_{t_0} = (h_s(t_0) - h_b(t_0))$. Therefore the third term on the right hand side of equation (4) describing the rate of change in ocean density over an area a and water column $h_s(t) - h_b(t)$ can be approximated by the discretised rate of change in steric sea level multiplied by the mean ocean density in the water column:

$$\int_{h_b(t)}^{h_s(t)} \frac{\partial \rho_o(t,h)}{\partial t} \mathrm{d}h \approx -\bar{\rho_o}(t) \frac{d\eta_s}{dt} \tag{S1}$$

References

Table S1. Ocean Mass Trend and the Rate of OBD from GRACE Products from Different Processing Centers.

	Center	$\dot{M}$ (mm/yr)	OBD (mm/yr)	OBD w/o buffer (mm/yr)
Mascons	JPL	1.63 ± 0.10	-0.11 ± 0.02	-0.07 ± 0.02
	GSFC	2.05 ± 0.15	-0.03 ± 0.01	-0.03 ± 0.01
	CSR	1.24 ± 0.11	-0.09 ± 0.02	-0.06 ± 0.02
Spherical Harmonics	CSR	1.65 ± 0.13	-0.14 ± 0.03	-0.09 ± 0.03
	GFZ	1.57 ± 0.12	-0.13 ± 0.02	-0.08 ± 0.02
	ITSG	1.26 ± 0.11	-0.09 ± 0.02	-0.06 ± 0.02
Mean		1.57 ± 0.30	-0.10 ± 0.05	-0.06 ± 0.05

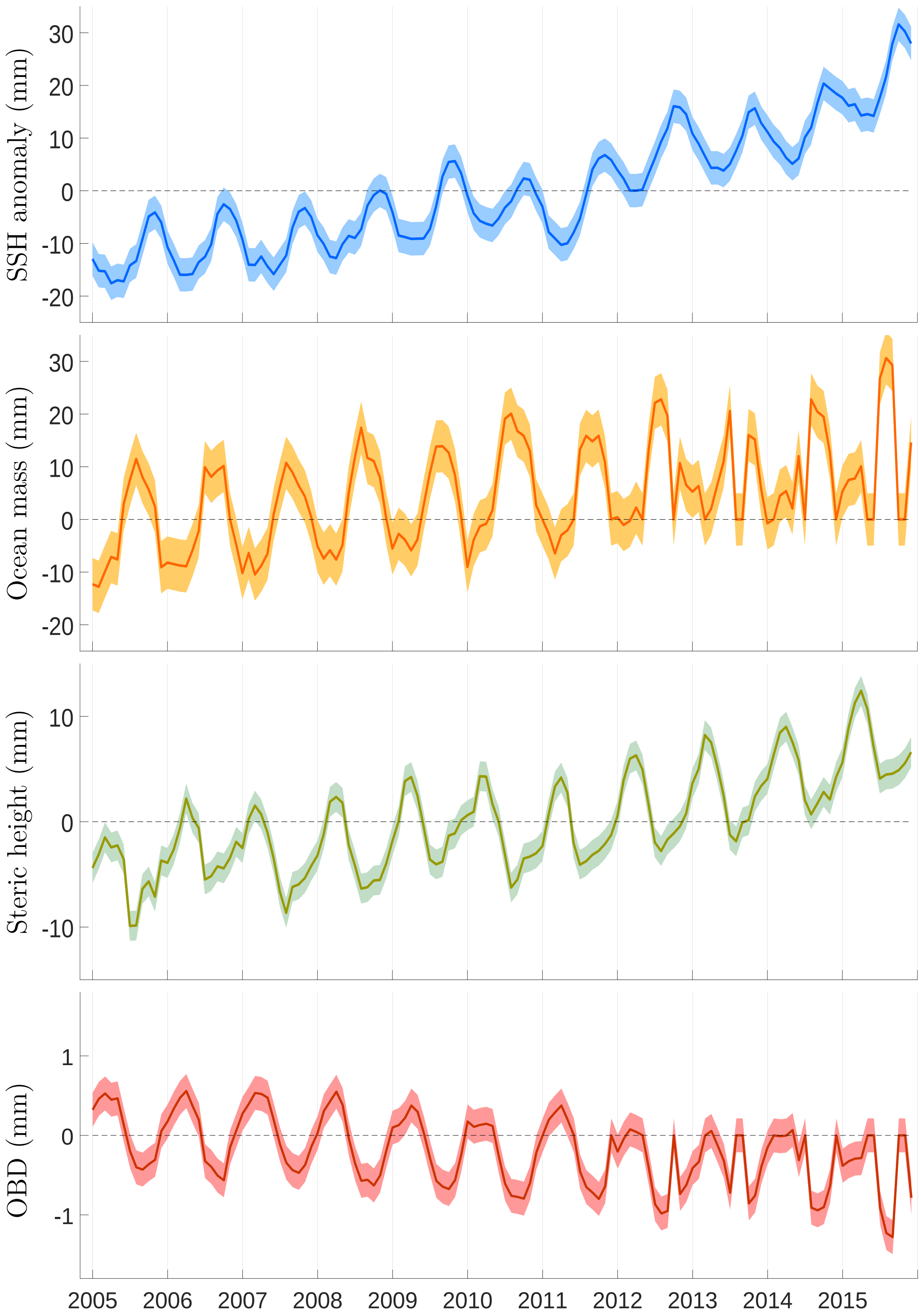

Figure S1. Time series of SLB components

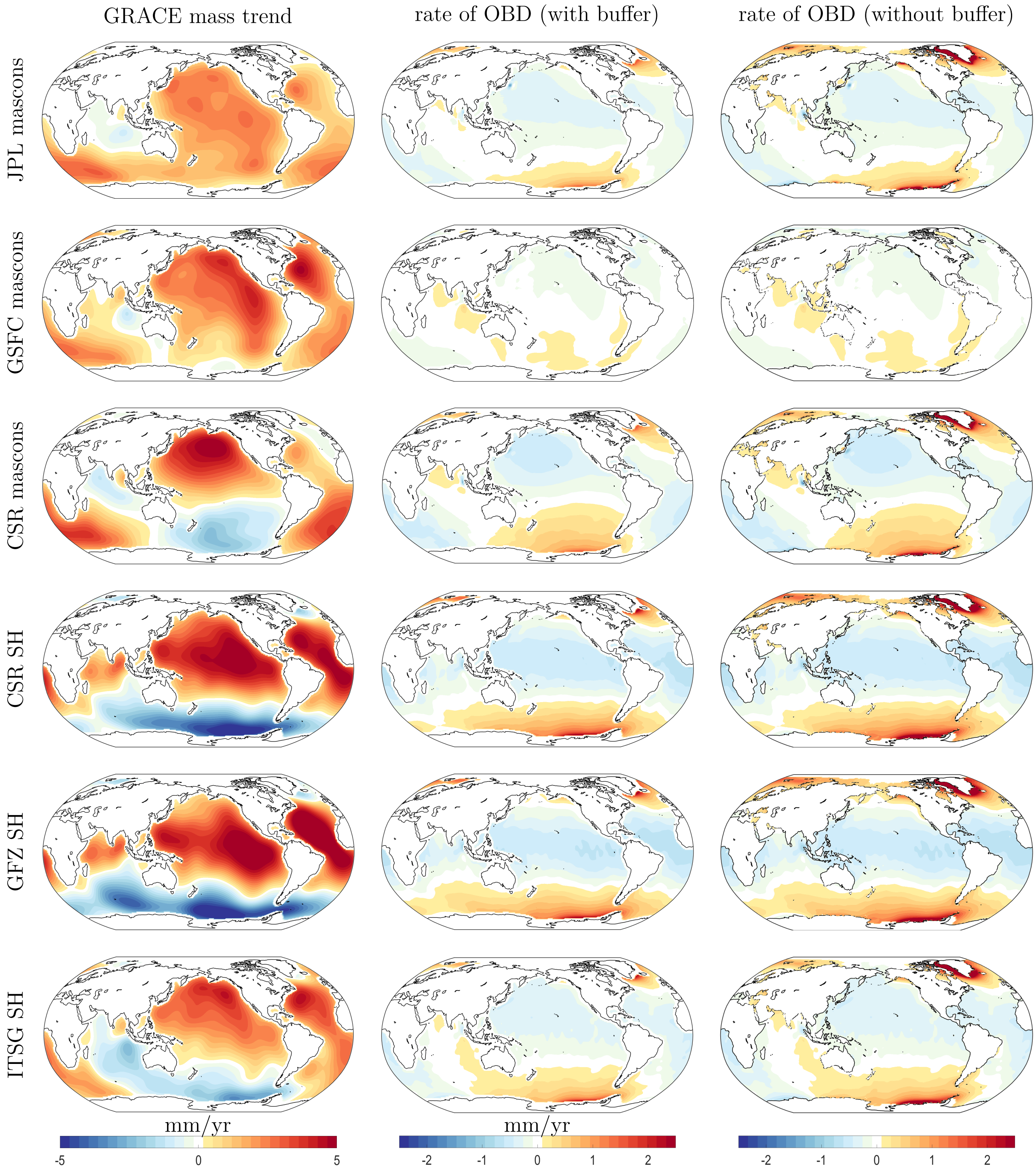

Figure S2. Maps of ocean mass trend and the corresponding rate of OBD from various GRACE products released by different centers. All the maps have been filtered with a 1000 km Gaussian filter and masked over land + 300 km buffer for visualization purposes.
