# Supplementary material for: Sea Level Budgets Should Account for Ocean Bottom Deformation
Source: Geophys Res Lett. 2020 Feb 11;47(3):e2019GL086492. doi: 10.1029/2019GL086492 (PMC7687171; doi:10.1029/2019GL086492)
Supplement: Supplementary file 3 — Figure S1 [file GRL-47-e2019GL086492-s003.pdf]

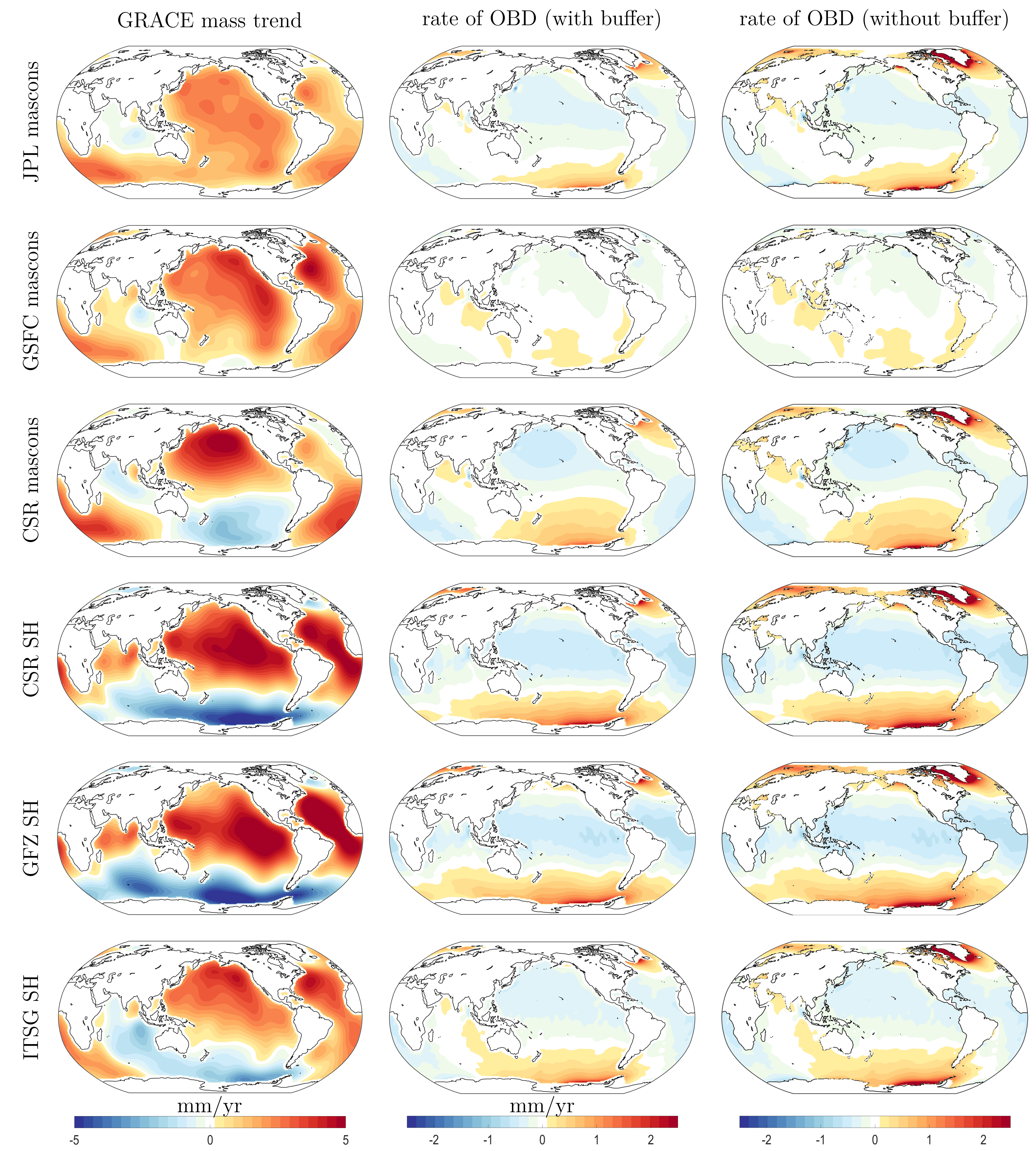

GRACE mass trend
rate of OBD (with buffer)
rate of OBD (without buffer)
JPL mascons
GSFC mascons
CSR mascons
CSR SH
GFZ SH
ITSG SH
mm/yr
-5
0
5
mm/yr
-2
-1
0
1
2
-2
-1
0
1
2
