# Supplementary figures and images for: Sea Level Budgets Should Account for Ocean Bottom Deformation
Source: Geophys Res Lett. 2020 Feb 11;47(3):e2019GL086492. doi: 10.1029/2019GL086492 (PMC7687171; doi:10.1029/2019GL086492)

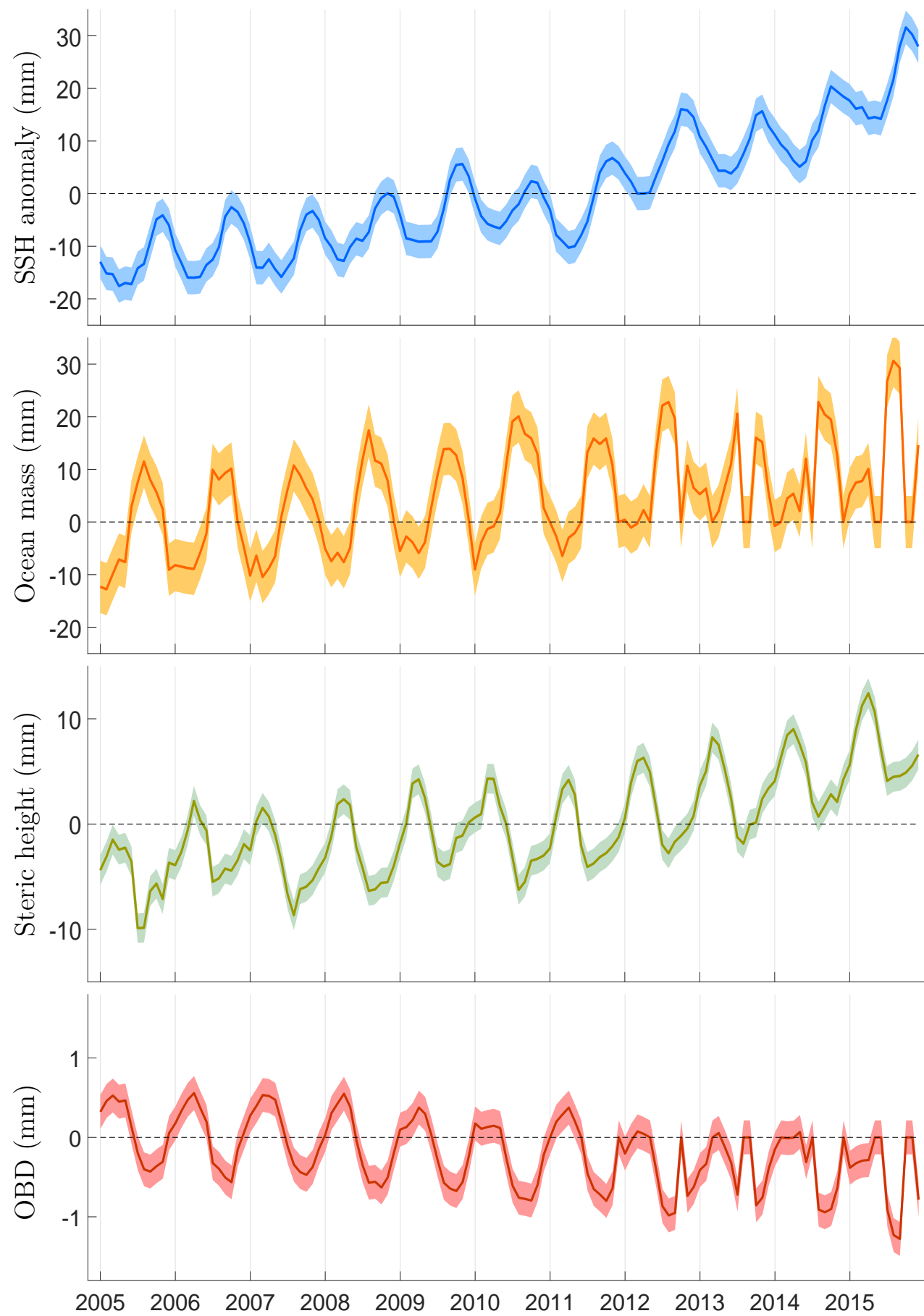

Supplement: Supplementary file 4 — Figure S2 [file GRL-47-e2019GL086492-s004.pdf]
